# Recurring Ethanol Exposure Induces Disinhibited Courtship in *Drosophila*

**DOI:** 10.1371/journal.pone.0001391

**Published:** 2008-01-02

**Authors:** Hyun-Gwan Lee, Young-Cho Kim, Jennifer S. Dunning, Kyung-An Han

**Affiliations:** 1 Department of Biology, Huck Institute Genetics Graduate Program, Pennsylvania State University, University Park, Pennsylvania, United States of America; 2 Department of Biology, Huck Institute Neuroscience Graduate Program, Pennsylvania State University, University Park, Pennsylvania, United States of America; 3 Department of Biology, Pennsylvania State University, University Park, Pennsylvania, United States of America; Centre National de la Recherche Scientifique, France

## Abstract

Alcohol has a strong causal relationship with sexual arousal and disinhibited sexual behavior in humans; however, the physiological support for this notion is largely lacking and thus a suitable animal model to address this issue is instrumental. We investigated the effect of ethanol on sexual behavior in *Drosophila*. Wild-type males typically court females but not males; however, upon daily administration of ethanol, they exhibited active intermale courtship, which represents a novel type of behavioral disinhibition. The ethanol-treated males also developed behavioral sensitization, a form of plasticity associated with addiction, since their intermale courtship activity was progressively increased with additional ethanol experience. We identified three components crucial for the ethanol-induced courtship disinhibition: the transcription factor regulating male sex behavior Fruitless, the ABC guanine/tryptophan transporter White and the neuromodulator dopamine. *fruitless* mutant males normally display conspicuous intermale courtship; however, their courtship activity was not enhanced under ethanol. Likewise, *white* males showed negligible ethanol-induced intermale courtship, which was not only reinstated but also augmented by transgenic White expression. Moreover, inhibition of dopamine neurotransmission during ethanol exposure dramatically decreased ethanol-induced intermale courtship. Chronic ethanol exposure also affected a male's sexual behavior toward females: it enhanced sexual arousal but reduced sexual performance. These findings provide novel insights into the physiological effects of ethanol on sexual behavior and behavioral plasticity.

## Introduction

Ethanol acts on multiple neural systems to produce diverse behavioral responses [1]–[3]. At low doses, ethanol induces euphoria and disinhibition whereas excessive consumption causes loss of motor control, sedation and sometimes fatality. A prominent euphoric response associated with ethanol in humans is sexual arousal. The enhanced arousal, in combination with the negative effect of ethanol on cognition, is believed to cause disinhibited sexual behavior, which possibly underlies risky sexual behavior such as unprotected sex and assaults associated with drinking [4]–[6]. The ethanol-associated sexual behavior appears to be due to expectancy (outcome based on learned anticipation) as well as pharmacological effects [5]; however, physiological evidence is lacking. Animal studies investigating ethanol's effects on sexual behavior have mainly focused on sexual performance, in which ethanol negatively affects copulatory behavior [7], [8]. Nonetheless, two studies specifically explored ethanol's effect on sexual motivation or arousal in male rats, but their findings are inconsistent [7], [9]. Therefore, the physiological underpinning of ethanol's effect on sexual arousal and disinhibition needs to be resolved.

The fruit fly *Drosophila melanogaster*, which offers vast genetic resources, tools and databases, is an excellent model to investigate the physiological mechanisms underlying behavior and has been adopted for studying addictive substances such as alcohol, cocaine, and methamphetamine [10]–[12]. Ethanol is naturally present in fermented fruits and cereals where fruit flies are usually found. Upon exposure to ethanol vapor, flies show increased locomotor activities and sedation [11]. Moreover, flies develop tolerance to the sedative effect of ethanol after a single exposure to high concentrations of ethanol or after a prolonged exposure to low concentrations, which is mediated by adaptive changes in brain activities [13], [14]. These biphasic and adaptive responses of flies to ethanol are strikingly similar to those of rodents and humans. This implies that ethanol affects the fly and mammalian nervous systems in a similar manner. In this study, we have explored whether recurring ethanol experience elicits behavioral changes in *Drosophila*. We report here that *Drosophila* males, upon repeated exposure to ethanol, not only developed tolerance to the sedative effect, but they also displayed active intermale courtship and behavioral sensitization to this effect. Moreover, the neural factor regulating male sexual behavior Fruitless^M^ (Fru^M^), the ABC guanine/tryptophan transporter White and the neuromodulator dopamine were crucial in the ethanol-induced courtship disinhibition.

## Results

### Tolerance development to the sedative effect of ethanol

**Figure 1:**
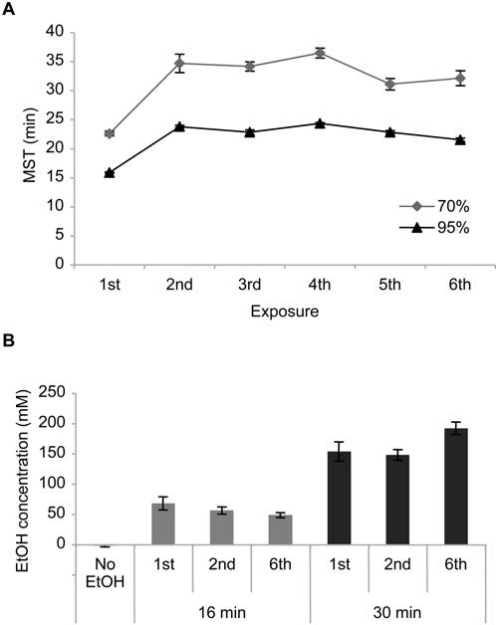
Effects of recurring ethanol exposure on wild-type *CS* males. (A) Sedation profile. Flies were exposed to ethanol vapor in 70% Flypub (diamond) or 95% Flypub (triangle). MST in 70% Flypub was higher than that in 95% Flypub on all exposures and recurring treatment in both ethanol concentrations increased MST. Two-way ANOVA revealed the significant effects of ethanol concentration and exposure, and a marginal interaction of two factors (concentration effect, *F_1,72_* = 383.4, *p*<0.0001; exposure effect, *F_5,72_* = 40.5, *p*<0.0001; interaction, *F_5,72_* = 2.7, *p* = 0.03; *n* = 7). *Post hoc* Tukey-Kramer tests revealed the significant difference of the 1^st^ from the other exposures in both ethanol concentrations. All data are reported as mean±standard error of the mean. (B) Ethanol concentrations. *CS* males were subjected to ethanol treatment for 1, 2 or 6 days (1^st^, 2^nd^, 6^th^) in 95% Flypub and ethanol contents were measured at 16 or 30 min after the onset of ethanol exposure. *CS* males without ethanol treatment (No EtOH) were used to measure the basal level. There was no significant difference in the ethanol contents of the males on the 1^st^, 2^nd^ and 6^th^ exposure at 16 min (ANOVA, *F_2,11_* = 1.75, *p* = 0.23, *n* = 4) or 30 min (*F_2,11_* = 3.98, *p* = 0.06, *n* = 4).

To investigate adaptive behavior associated with recurring exposure to ethanol, we developed a novel apparatus Flypub. Flypub is made of a plastic chamber with a clear ceiling for videotaping behavior and an open bottom for administering ethanol. We exposed fully mature (4 to 5 day-old) wild-type *Canton-S* (*CS*) males to intoxicating doses of ethanol once a day for 6 days in Flypub. Prior to ethanol exposure, flies were acclimated to the chamber and then the whole unit was gently placed on a Petri dish containing a cotton pad applied with 70% or 95% ethanol. While slowly exposed to ethanol vapor, flies showed sequential behavioral changes: they became hyperactive (fast walking), lost motor control (infrequent movements and frequent falls during walking), and then were sedated (lying on their back). On the first exposure to ethanol vapor in 95% Flypub, all male flies became sedated within 24 min with a mean sedation time (MST) of approximately 16 min, whereas it took longer with 70% ethanol (MST ∼23 min; Figure 1). The flies on the second exposure showed a similar activity profile with a delayed sedation time (∼24 min MST with 95% and ∼35 min with 70%), indicating tolerance development to the sedative effect of ethanol. The tolerance level, as measured by MST, did not change significantly with additional ethanol exposure on consecutive days (Figure 1). It is difficult to compare MST observed in Flypub with that reported in other studies, which employ diverse devices, conditions and parameters. Nonetheless, the mean elution time (MET) of wild-type flies exposed to humidified ethanol vapor (50/45 ethanol/air flow) in the 4 foot-long inebriometer is ∼20 min with 40% and 25% increases in MET on the second exposure at 4 h and 24 h intervals, respectively [13]. When measured in the perforated 50 mL Falcon tube with 50% ethanol vapor, on the other hand, MET (time for immobilizing 50% of flies) is ∼16 min with 100% increase in MET on the second exposure at a 4 h interval [15]. Thus, MST on the first exposure and tolerance levels on the second exposure measured in 95% (16 min; 50%) and 70% (23 min; 52%) Flypub are within the range observed in other studies. Notably, the ethanol concentrations measured at 16 or 30 min after administering ethanol were comparable in the males subjected to ethanol treatment for 1, 2 and 6 days (Figure 1B). Therefore, tolerance developed upon repeated ethanol exposure is not due to altered ethanol absorption or metabolism, but likely due to adaptive changes in neural activities.

### Recurring ethanol exposure induces intermale courtship and behavioral sensitization

**Figure 2:**
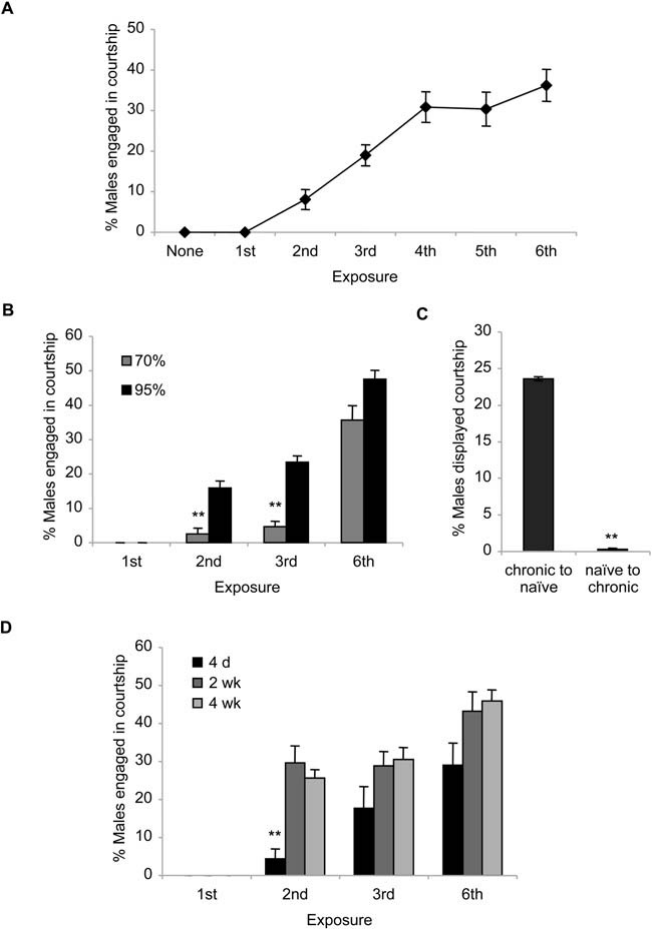
Effects of recurring ethanol exposure on courtship behavior of *CS* males. (A) The percentage of males engaged in intermale courtship progressively increased upon additional ethanol treatments in 95% Flypub. Least squares regression showed the significant effect of exposure (*r^2^* = 0.68, *p*<0.0001, *n* = 10). (B) *CS* males subjected to daily ethanol exposure in 70% Flypub exhibited the exposure-dependent increase in intermale courtship but at the significantly reduced levels compared to those challenged with 95% ethanol. Two-way ANOVA revealed the significant effects of ethanol concentration, exposure, and interaction (concentration effect, *F_1,24_* = 54.02, *p*<0.0001; exposure effect, *F_3,24_* = 138.2, *p*<0.0001; interaction, *F_3,24_* = 6.7, *p* = 0.0019; *n* = 4). *Post hoc* two-tailed Student *t*-test showed the significant difference of the courtship scores on the 2^nd^ (*p*<0.005) or 3^rd^ exposure (*p*<0.0005) (marked by double asterisks). (C) The chronic-ethanol-treated males displayed active courtship toward the decapitated previously-ethanol-naïve males under the influence of ethanol (chronic to naïve), whereas the ethanol-naïve males, on the 1^st^ ethanol exposure, displayed negligible courtship toward the decapitated chronic-ethanol-exposed males (naïve to chronic). Two-tailed Student *t*-test showed a significant difference (*p*<0.0001, *n* = 6, marked by double asterisks). (D) Two or 4 wk-old *CS* males exhibited the increased levels of intermale courtship compared to 4 d-old males when tested in 95% Flypub. Two-way ANOVA revealed the significant effects of age and exposure, and a marginal interaction (age effect, *F_2,36_* = 16.6, *p*<0.0001; exposure effect, *F_3,36_* = 61.7, *p*<0.0001; interaction, *F_6,36_* = 2.58, *p* = 0.035; *n* = 4). Tukey-Kramer tests showed that the intermale courtship activities of 2 and 4 wk-old males were significantly different from that of 4 d-old males on the 2^nd^ exposure (marked by double asterisks).

Upon daily ethanol treatments, *CS* males showed distinct sexual behavior. Typically, *Drosophila* males vigorously court females that have attractive pheromones with the courtship ritual comprising a sequential act of following, tapping the female's abdomen, wing vibration (courtship song), licking the female's genitalia, and attempted copulation, which eventually leads to copulation [16], [17]. *Drosophila* males, on the contrary, rarely exhibit active courtship toward other mature males [18], which we also observed in the absence of ethanol or on the first exposure to ethanol (Figure 2A, Movie S1). Occasionally, a male attempted to court another male but quickly moved away. Also, a male courtee strongly rejected a courting male (Movie S1). Under the influence of ethanol on the second and subsequent ethanol treatment, however, *CS* males actively courted other males in the ritual similar to that shown toward females, which represents disinhibited courtship.

Notably, the ethanol-induced courtship was dynamic (Movie S1): courtship duration of each pair or chain (courters courted by courtees) ranged from a couple of seconds to minutes and new courtship pairs or chains were continuously formed. These dynamic courtship activities typically lasted for 5 to 10 min before the flies began loosing motor control and became sedated. Moreover, courtship chains with the length ranging from 3 to 5 were frequently noticeable on the third and subsequent ethanol challenges while courtship pairs were dominant on the second exposure. To quantify the ethanol-induced courtship activity, we scored the percentage of males engaged in active courtship during a 30 sec period and used an average of 10 consecutive periods (total 5 min) to represent a percentage of courtship for each group. The percentage of *CS* males engaged in intermale courtship increased with recurring experience of ethanol, reaching a plateau on the 4^th^ exposure with 95% ethanol (Figure 2A), whereas the males subjected to daily mock treatment did not display any intermale courtship (*n* = 6). Likewise, no intermale courtship activity was detected when the chronic-ethanol-treated males (daily ethanol for 5 or 6 days) were examined in Flypub in the absence of ethanol (*n* = 6 each). Therefore, the observed intermale courtship requires physiological actions of ethanol. Similar increases in the courtship activity were observed with 70% ethanol, but with lesser extents on the second and third exposures (Figure 2B). These observations together indicate that *Drosophila* males develop behavioral sensitization to the ethanol's effect on courtship disinhibition.

We assessed whether the ethanol-induced intermale courtship is due to changes in aversive male pheromones, which may become attractive upon repeated ethanol exposure. If this were the case, ethanol-treated males would not court ethanol-naïve males while ethanol-naïve males would actively court ethanol-treated males. To test this, the males treated with daily ethanol for 5 days (chronic-ethanol-treated males) were subjected to ethanol exposure in the presence of the decapitated previously-ethanol-naïve males. In another set of experiments, the ethanol-naïve males were housed with the decapitated chronic-ethanol-treated males and tested for their courtship under the influence of ethanol. The chronic-ethanol-treated males displayed active courtship toward the previously-ethanol-naïve males; however, the courtship activity of the naïve males toward the ethanol-treated males was negligible (Figure 2C). Thus, the ethanol-induced intermale courtship is unlikely caused by altered male pheromones.

A salient effect of ethanol is cognitive impairment [5], [19], which may account for the disinhibited courtship of sexually aroused males under the influence of ethanol. Thus, we reasoned that the aged males whose cognitive capacity is reduced [20] might exhibit enhanced ethanol-induced courtship disinhibition. When 2 or 4 wk-old males were subjected to daily ethanol exposure, a significantly higher percentage of males showed intermale courtship on the second ethanol exposure compared to 4 day-old males while the difference was less apparent on subsequent exposures (Figure 2D). This implies that certain aging-sensitive activities may be related to the ethanol-induced adaptive changes underlying courtship disinhibition. Taken together, recurring ethanol administration induced conspicuous intermale courtship, which represents disinhibited sexual behavior and entails certain adaptive changes prompted by initial ethanol exposure. The significant increases in this activity in the absence of concurrent increases in tolerance imply distinct mechanisms underlying behavioral sensitization to the disinhibition effect and tolerance to the sedative effect of ethanol.

### Fruitless is crucial for the ethanol-induced intermale courtship

**Figure 3:**
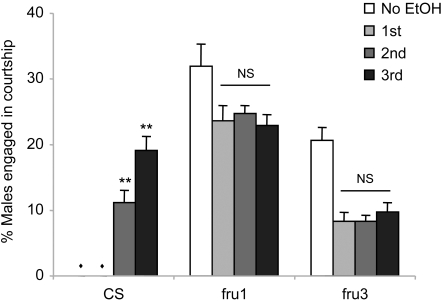
Effects of chronic ethanol on courtship behavior of *fru* males. *fru^1^* and *fru^3^* males showed vigorous intermale courtship in Flypub without ethanol (No EtOH). Upon daily ethanol treatments in 95% Flypub, their courtship activities did not change with additional ethanol treatment while the courtship levels under ethanol were lower than those without ethanol (ANOVA: *fru^1^*, *F_3,27_* = 3.24, *p* = 0.0396, Tukey-Kramer showed a significant difference between No EtOH and 3^rd^ EtOH; *fru^3^*, *F_3,27_* = 16.94, *p*<0.0001, No EtOH was significantly different from the others by Tukey-Kramer; *n* = 7). NS, not significant. *CS* males tested together with *fru* males as a control showed negligible intermale courtship in the absence of ethanol and on the 1^st^ ethanol exposure (marked by diamonds); however, their courtship activities were enhanced under the influence of ethanol on the 2^nd^ and 3^rd^ ethanol treatments (*F_3,27_* = 42.86, *p*<0.0001; double asterisks, significant difference by Tukey-Kramer).

Genetic alterations in somatic sex development are known to cause intermale courtship in *Drosophila* [21]. The studies described here, in contrast, reveal recurring ethanol exposure as a post-developmental factor affecting male sexual behavior. We asked whether ethanol affects the brain activity in the manner that the altered brain development causes intermale courtship. If this were the case, ethanol would further enhance the intermale courtship activity of *fruitless* (*fru*) males defective in Fru^M^, a neural sex determination factor. To test this, we employed two *fru* mutant alleles *fru^1^* and *fru^3^*, which have abnormal expression of male-specific Fru^M^ in the central nervous system. *fru^1^* males have an inversion break point 3.3 Kb upstream of the sexually dimorphic P1 promoter, causing altered Fru^M^ expression: subpopulations of Fru^M^ neurons have either less or undetectable Fru^M^ while numerous non-Fru^M^ neurons display ectopic Fru^M^ expression [22]. On the other hand, *fru^3^* males with a transposon insertion at the second intron in the *fru* gene have undetectable Fru^M^ expression [22]. Both *fru^1^* and *fru^3^* males exhibit active intermale courtship [22], which we also observed in Flypub without ethanol (Figure 3). When subjected to daily ethanol exposure, the courtship levels of *fru^1^* and *fru^3^* males were decreased on the first exposure, which remained unchanged on subsequent exposures (Figure 3). While the inability for *fru* males to increase levels of intermale courtship upon recurrent ethanol exposure could be due to the behavioral ceiling effect, the intermale courtship level of *fru^3^* males is significantly lower than that of CS males on the third exposure (two tailed Student *t*-test, p<0.001). This suggests that normal physiological function of Fru^M^ or the male-specific neural system established by Fru^M^ is crucial for the ethanol-induced intermale courtship.

### ABC transporter White is involved in the ethanol-induced courtship disinhibition

**Figure 4:**
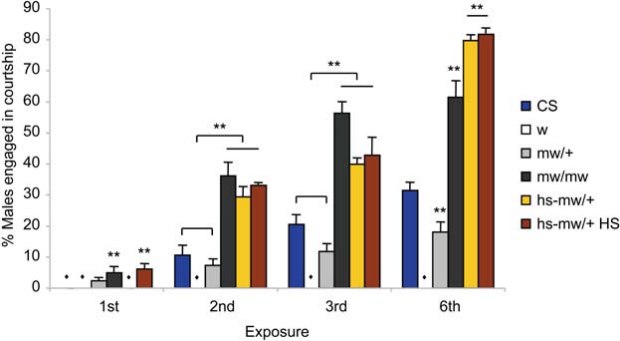
Ethanol-induced courtship and sedation of *w* and transgenic *w* males. *w^1118^* males did not display any discernible ethanol-induced intermale courtship upon recurring ethanol treatment, which was reversed by transgenic *w* expression in *mw^+^*/+, *mw^+^*/*mw^+^*, and *hs-mw^+^/+* without (*hs-mw^+^/+*) and with heat shock (*hs-mw^+^/+* Hs). Diamonds denote no courtship observed. General linear model revealed the significant effects of exposure, genotype, and interaction (exposure effect, *F_3,167_* = 266.85, *p*<0.0001; genotype effect, *F_5,167_* = 181.04, *p*<0.0001; double asterisks, significant difference by Tukey-Kramer tests in each exposure; interaction, *F_15,167_* = 28.23, *p*<0.0001; *n* = 3–11).

We surveyed pre-existing fly mutants to investigate the cellular mechanism underlying the ethanol-induced courtship disinhibition and found a commonly used strain *white^1118^* (*w^1118^*) displaying negligible intermale courtship upon recurring ethanol administration with 70% (*n* = 6) or 95% ethanol (*n* = 32; Figure 4; Movie S2). A similar result was obtained with the independent allele *w^1^* (*n* = 6 for both 70% and 95% ethanol), indicating a strong association of the observed phenotype with the *w* mutation. The *w* gene encodes an ABC transporter for guanine and tryptophan that are crucial not only for eye pigmentation but also for dopamine (guanine) or serotonin (guanine and tryptophan) biosynthesis [23]. *w* males whose eyes are depigmented have a normal capacity to detect light as judged by their strong preference for a lighted to a dark area (*n* = 8; data not shown); nonetheless, the *w* male's deficient intermale courtship could be due to their potential visual problem. To explore this possibility, we administered daily ethanol to *CS* males under infrared light, in which flies can't see. In all exposures up to the 6^th^, *CS* males did not show any intermale courtship (*n* = 6). Thus, visual input is indispensable for the ethanol-induced intermale courtship and a potential visual anomaly of *w* males may attribute to their deficient response.

To test whether the *w^1118^* male's phenotype could be rescued by reinstating White expression, we employed two independent lines f06195 and PHSBJb_3_, which carry the transgenic *mini-white* (*mw^+^*) gene in the transformation vectors piggyBAC and P-element, respectively [24], [25]. The *mw^+^* gene represents a *w* genomic clone including 300 bp 5′ and 630 bp 3′ endogenous regulatory sequence but lacking most of the first intron [26]. In addition, the *mw^+^* gene in PHSBJb_3_ is under the influence of neighboring heat-shock promoter, which induces over-expression of *mw^+^* at 37°C [25]. The heterozygous f06195 and PHSBJb_3 _males in the *w^1118^* mutant background (*mw^+^/+* and *hs-mw^+^/+;* Figure 4; Movie S2) exhibited intermale courtship under the influence of ethanol in an exposure-dependent manner. This indicates that White, the protein absent in the *w* mutant, is essential for the ethanol-induced courtship disinhibition.

Interestingly, the ethanol-induced courtship activities of homozygous f06195 (*mw^+^/mw^+^*) and heterozygous PHSBJb_3_ (*hs-mw^+^/+*) males with or without heat shock (incubation at 37°C for 1 h once a day for 3 days prior to ethanol exposure) were higher than that of *CS* males in all exposures except for the first (Figure 4). On the first exposure, significant levels of intermale courtship were detected in *mw^+^/mw^+^* males and *hs-mw^+^/+* males with heat shock (*hs-mw^+^/+* HS) (Figure 4A) while the same heat treatment did not induce intermale courtship in *CS* and *w* males (*n* = 6). The *w* gene is normally expressed in the eye pigment cells and the brain [27], in which its expression pattern is unknown. Notably, the eye colors of all transgenic *mw^+^* males were lighter than that of *CS* males and the males expressing *mw^+^* in the *CS* (*w+*) background displayed enhanced ethanol-induced intermale courtship levels similar to those in the *w* mutant background (data not shown). Therefore, while deficient White in the eye appears to primarily account for the poor ethanol-induced courtship in *w* males, over- or mis-expressed White may enhance intermale courtship under ethanol possibly by acting on the cellular pathway(s) underlying courtship disinhibition. Taken together, the ABC guanine/tryptophan transporter White is essential for the ethanol-induced courtship disinhibition and its ectopic or increased expression leads to a high propensity to this behavior.

### Dopamine is essential for ethanol-induced courtship disinhibition

**Figure 5:**
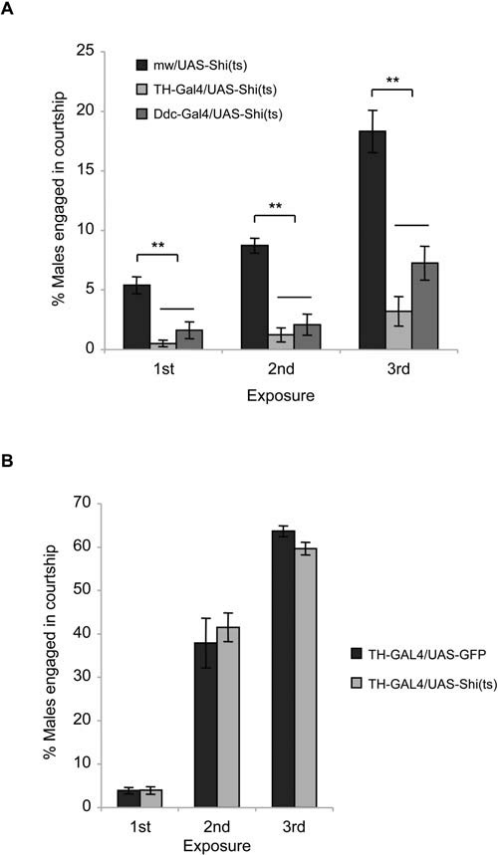
Effects of inhibited dopamine neuronal activities on the ethanol-induced courtship. (A) *TH-GAL4/UAS-Shi^ts^* and *DDC-GAL4/UAS-Shi^ts^* males, when subjected to daily ethanol exposure at 32°C to block synaptic output of dopamine neurons, displayed drastically reduced intermale courtship compared to the control *mw^+^/UAS-Shi^ts^* males on all exposures (1^st^ exposure, *F_2,21_* = 18.24, *p*<0.0001; 2^nd^, *F_2,21_* = 35.26, *p*<0.0001; 3^rd^, *F_2,21_* = 27.02, *p*<0.0001; *n* = 7–8). Double asterisks, significant difference by Tukey-Kramer tests. (B) *TH-GAL4/UAS-Shi^ts^* males, when subjected to daily ethanol exposure at room temperature, showed intermale courtship activities comparable to those of the control *TH-GAL4/UAS-GFP* males, indicating that Shi^ts^ expressed in dopamine neurons without the dominant negative activity has no effect on the ethanol-induced intermale courtship (Student *t*-test on all exposures, *p*>0.5, *n* = 7).

Ethanol acts on multiple neural systems; however, the altered intermale courtship activities of *w* and *mw^+^* males point to dopamine and serotonin as primary culprits for the ethanol-induced courtship disinhibition. To test this, we employed the GAL4/UAS system [28] and temperature-sensitive dominant negative Dynamin Shi^ts^ [29] to manipulate dopamine neuronal activities. At 30°C or higher restrictive temperature, Shi^ts^ inhibits endocytosis and thus blocks synaptic output. Transgenic TH (*tyrosine hydroxylase* enhancer)-GAL4 or DDC (*dopa decarboxylase* enhancer)-GAL4 flies express the transcription factor GAL4 in dopamine or dopamine and serotonin neurons, respectively, to activate expression of the gene (e.g. Shi^ts^) downstream of UAS [30], [31]. Thus, we tested *TH-GAL4/UAS-Shi^ts^* and *DDC-GAL4/UAS-Shi^ts^* males to recurring ethanol exposure at the restrictive temperature, under which condition synaptic output of dopamine and dopamine/serotonin neurons, respectively, is inhibited. Since all transgenes are tagged with *mw^+^* as an *in vivo* transformation marker, *mw^+^/UAS-Shi^ts^* males were used as a control to match the *mw^+^* copy number. When subjected to daily ethanol exposure at 32°C, both *TH-GAL4/UAS-Shi^ts^* and *DDC-GAL4/UAS-Shi^ts^* males showed significantly reduced intermale courtship activities compared to *mw^+^/UAS-Shi^ts^* males on all exposures examined (Figure 5A). When tested at room temperature, on the contrary, *TH-GAL4/UAS-Shi^ts^* males displayed the ethanol-induced intermale courtship activities comparable to those of the control males (Figure 5B). Thus, Shi^ts^ in the absence of dominant negative activities has no effect on the ethanol-induced intermale courtship. These observations indicate that synaptic output of dopamine neurons is required for the ethanol-induced courtship disinhibition.

### Ethanol affects male sexual behavior toward females

**Figure 6:**
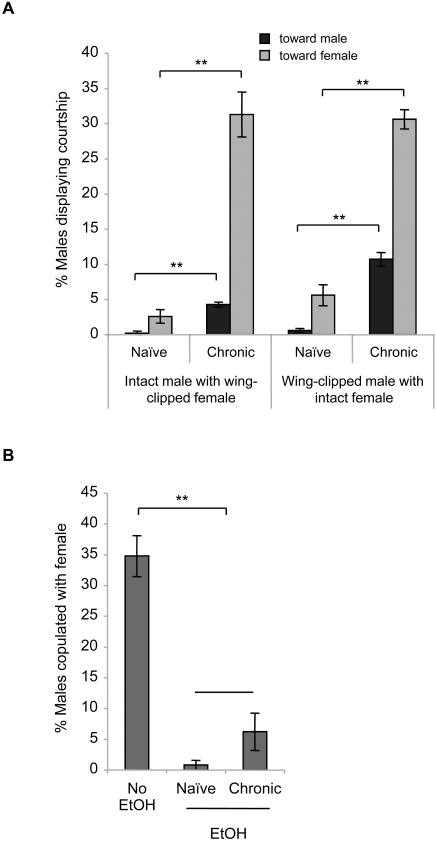
Effects of ethanol on male sexual behavior toward females. (A) Courtship. The wings of *CS* males or females were clipped to distinguish the sex. The ethanol-naïve or ethanol-treated males were housed with an equal number of virgin females and subjected to ethanol exposure in 95% Flypub. Two-factor ANOVA revealed the significant effects of partner, exposure, and interaction in both sets of experiments (intact male with wing-clipped female: partner effect, *F_1,27_* = 75.8, *p*<0.0001; exposure effect, *F_1,27_* = 93.9, *p*<0.0001; interaction, *F_1,27_* = 53.2, *p*<0.0001; *n* = 7. Wing-clipped male with intact female: partner effect, *F_1,27_* = 120.8, *p*<0.0001; exposure effect, *F_1,27_* = 239.5, *p*<0.0001; interaction, *F_1,27_* = 43.2, *p*<0.0001; *n* = 7). Double asterisks, significant difference by planned Student *t*-tests. (B) Copulation. *CS* males under the influence of ethanol on the 1^st^ and 6^th^ exposures displayed significantly reduced copulation with virgin females (ANOVA, *F_2,20_* = 47.7, *p*<0.0001; double asterisk, significant difference by Tukey-Kramer; *n* = 7).

Courtship of *Drosophila* females is usually passive. Upon daily ethanol treatment, females did not show courtship toward other females (*n* = 6). Thus, the effect of ethanol on homosexual courtship is specific to males. We next addressed whether the ethanol-induced intermale courtship is attributable to altered sexual orientation of males. If this were the case, chronic-ethanol-treated males would prefer courting males to females. To test this, the age-matched, ethanol-naïve and chronic-ethanol-treated (daily ethanol treatment for 5 days) *CS* males were subjected to ethanol exposure in the presence of *CS* virgin females. In this experiment, the wings of either males or females were clipped to distinguish the sex. Typically, males in the absence of ethanol vigorously court virgin females and readily engage in copulation that lasts approximately 20 min [32]. To differentiate sexual behavior affected by ethanol from basal behavior, males and females were acclimated in separate compartments in Flypub and mixed together immediately after ethanol administration. Under the influence of ethanol, the previously ethanol-naïve males displayed a small, but significant, courtship activity toward females and negligible courtship toward males (Figure 6A). In contrast, the chronic-ethanol-treated males exhibited dramatically increased courtship toward females as well as males; nonetheless, a significantly larger number of males courted females than males. It was also noticeable that the courting males routinely changed a courtship partner from a male to a female and vice versa. These observations indicate that recurring ethanol experience does not change sexual orientation of males; rather, it enhances sexual arousal and disinhibition.

Contrary to the effect of chronic ethanol, the initial ethanol experience has a negative effect on male courtship behavior toward females. Without ethanol, males actively courted females, which were typically followed by copulation (Figure 6B); however, the courtship activity was drastically diminished under the influence of ethanol on the first exposure (Figure 6A). Ethanol also affected sexual performance. In the absence of ethanol, approximately 35% of males copulated with females, whereas only a small percentage of the ethanol-naïve or chronic-ethanol-treated males copulated with females under the influence of ethanol (Figure 6B). Thus, both initial and recurring ethanol treatments have a negative effect on copulation.

## Discussion

In this report, we have shown that *Drosophila* males, when subjected to repeated exposure to ethanol, display disinhibited courtship toward other mature males, which is enhanced by additional ethanol challenges. Ethanol affects various aspects of sexual behavior in humans: it is known to impair sexual performance and to enhance sexual arousal or motivation [4], [5]. Moreover, disinhibited sexual behavior is highly associated with alcohol consumption; however, the physiological support for this notion is largely lacking and a suitable animal model to address this issue is instrumental. In a rodent model, a low dose of ethanol reinstates copulatory behavior of the male rats that have been repeatedly trained to suppress their sexual response to unreceptive females [33]. Unfortunately, the study did not distinguish whether the disinhibition effect is on sexual arousal/motivation or sexual performance. Scott et al. have followed up this issue and failed to observe disinhibited sexual motivation [9]. The findings described here provide for the first time unambiguous evidence for disinhibited sexual arousal induced by ethanol and behavioral sensitization to this effect.

Disinhibited homosexual courtship has been previously reported on genetic and transgenic mutants. In particular, homosexual courtship is obvious in transgenic males expressing female Transformer^F^ (a key component of somatic sex determination) or transgenic females expressing Fru^M^ (a downstream target of Transformer) in distinct neuronal populations, and *fru* males with aberrant Fru^M^ expression [34]–[38]. These studies indicate that sexual orientation and behavior are controlled by the brain circuitry established by Fru^M^ during development. The findings described here, on the other hand, unveil a post-developmental experience of recurring ethanol as a key factor affecting sexual behavior of wild-type males. In the absence of ethanol, *fru^1^* and *fru^3^* males with altered or undetectable Fru^M^ expression, respectively, display characteristic intermale courtship, whose levels were slightly (*fru^1^*) or significantly (*fru^3^*) reduced under the influence of ethanol. The male's courtship toward the female is typically initiated by visual and pheromonal input. The ethanol-induced intermale courtship, on the other hand, appears to depend largely on visual input and less on pheromones since CS males exposed to ethanol under infrared light show negligible intermale courtship as noted above. It is conceivable that reduced levels of intermale courtship observed in *fru* males under ethanol could be attributable to compromised pheromonal input. Alternatively, certain intermale courtship activities associated with abnormal Fru^M^ may be negatively affected by ethanol. It is yet unclear how aberrant Fru^M^ expression causes increased intermale courtship in *fru^1^* and *fru^3^* males and enhanced understanding of this process should help resolve this issue. Remarkably, repeated ethanol exposure has no effect on enhancing intermale courtship in *fru* males. Fru^M^ is normally expressed not only during development but also at the adult stage [22]. Thus, disinhibited courtship induced by ethanol may recruit a physiological Fru^M^ function or a Fru^M^ neural circuit established during development or both. Future studies of additional *fru* alleles or transheterozygotes with different lesions in the *fru* gene [39] along with temporally and spatially controlled transgenic manipulation of Fru^M^ expression should be instrumental to unravel the mechanism by which Fru^M^ mediates the ethanol-associated courtship disinhibition.

Ectopic or increased White expression is previously shown to trigger intermale courtship in the transgenic males carrying *mw^+^* gene under the control of heat shock promoter [25]. Indeed, PHSBJb_3_ employed in our study was one of the lines used in the previous study wherein homozygous PHSBJb_3_ males in the *Df(1)w67c2, yellow* genetic background exhibit intermale courtship after heat treatment in a densely populated culture bottle. Under the experimental condition (a low density population) and the genetic background (*w^1118^*) used in our study, they did not show a significant level of intermale courtship in the absence of ethanol with or without heat shock. Besides its function in body color pigmentation, Yellow in the brain is crucial for male sex development [40]. This implies that a combined action of *yellow* mutation and ectopic White in PHSBJb_3_ males may attribute to the enhanced intermale courtship observed in the previous study. Similarly in our study, the males with transgenic White expression were more susceptible to the ethanol-induced disinhibited courtship compared to *CS* males. While White has been extensively used as an eye color marker, several studies indicate the significant roles of White in the central nervous system. Notably, *w* mutants subjected to submaximal training learn poorly in operant heat-box conditioning, in which flies learn to avoid entering a hot temperature-associated chamber, whereas they learn better in classical olfactory conditioning, in which flies lean to avoid the odor associated with electric shock [41]. *w* mutants also show a reduced sensitivity to general anesthetics enflurane and halothane [27]. These studies demonstrate the distinct roles of White in various types of behavioral plasticity and anesthesia beyond its function in the eye. While it remains to be resolved whether and how White in the brain is involved in ethanol-induced courtship disinhibition, it is at least conceivable that a major action of over-/mis-expressed White is to inhibit the neural system mediating intermale courtship suppression, potentiating the ethanol-induced courtship disinhibition.

Regarding the cellular mechanism underlying the ethanol-induced courtship disinhibition, the biochemical functions of the White ligands guanine and tryptophan suggest dopamine and serotonin as key components. This notion is supported by the observations that certain polymorphisms in *hW,* the human homologue of *w* encoding ABCG1, are linked in males to panic and mood disorders, which are associated with abnormal monoamine functions [42], [43]. Consistently, our results reveal an essential role of dopamine neuronal activities (and presumably released dopamine) in courtship disinhibition induced by ethanol. Dopamine plays crucial roles in mediating the locomotor-activating, rewarding, and behavioral sensitization effects of ethanol in mammals [2], [44]. Indeed, ethanol intake increases dopamine levels in several brain areas and adaptive changes in the dopamine transporter and receptors are associated with alcoholism in humans and rodent models [2], [45]–[47]. Thus, dopamine is a key neuromodulator mediating the pleiotropic effects of ethanol, which is processed by various dopamine receptors in distinct neural systems in mammals, and similar mechanisms may underlie the ethanol-induced behaviors in flies.

Dopamine is also implicated in sexual motivation or arousal in humans, rodents, and flies [12], [48]–[50]. Particularly in flies, transgenic males overexpressing vesicular monoamine transporter in DDC-GAL4 neurons or wild-type males fed with methamphetamine display enhanced courtship toward females [12], [50]. While none of the previous studies in flies and mammals have specifically addressed the dopamine's role in disinhibited sexual behavior, it is conceivable that altered dopamine activities induced by ethanol may be responsible not only for enhancing sexual arousal but also for impairing cognition, causing disinhibited courtship. Indeed, dopamine is involved in numerous cognitive processes including attention, goal-directed behavior, and learning and memory [51], [52]. Five receptor subtypes (D1-5) mediate diverse dopamine functions in mammals. Similarly, *Drosophila* has D1, D2 and D5 receptors and we have recently identified the distinct functions of the D1 receptor dDA1 for punishment and reward memory formations in olfactory conditioning [53]. Future studies uncovering dopamine receptor subtypes involved in an arousal or cognitive aspect of disinhibited courtship should provide substantial insights on the underlying cellular mechanism.

The deficient ethanol-induced courtship in the males with defective Fru^M^ or dopamine neuronal activities suggests that Fru^M^ and dopamine systems may be functionally connected to each other for regulating male sexual behavior. One pathway could be for dopamine to modulate the Fru^M^ neural circuit. Interestingly, both Fru^M^ and dDA1 are highly enriched in the mushroom body neurons projecting to the gamma lobe [37], [38], [54]. It is possible that dopamine, upon binding to dDA1, may play a role in modifying the gamma lobe function established by Fru^M^ for courtship disinhibition. Alternatively, Fru^M^ may regulate dopamine neuronal activities. This could occur through direct or indirect interactions of Fru^M^ and dopamine neurons since both neuronal populations project to many overlapping brain areas [37], [55]. It is noteworthy that a dopamine neuron in each hemisphere, which projects to the anterior commissure and anterior brain areas, is positive for Fru^M^ expression (our unpublished observation). Future studies clarifying the functional interaction of Fru^M^ and dopamine activities are of great importance to delineate the cellular mechanism underlying the ethanol-induced courtship disinhibition.

The observations described here also reveal the dual effects of ethanol on the heterosexual courtship activity, which is reduced under initial ethanol exposure but enhanced upon chronic treatment. The effect of acute ethanol on sexual arousal or motivation has previously been addressed by two studies in rats. When tested for the operant lever-pressing response to get access to receptive females, the ethanol-injected male rats show increased latencies, implying attenuated sexual motivation [9]. On the other hand, the ethanol-injected male rats show increased frequencies to change platforms prior to encountering receptive females, suggesting enhanced sexual motivation [7]. While our study supports the former, ethanol's effect on sexual arousal/motivation may depend on multiple factors including measurement methods and routes of ethanol administration. Nonetheless, a consistent effect of ethanol on heterosexual behavior observed in our studies of flies and the previous findings in rats [7], [8] and humans [5] is the inhibitory effect on sexual performance. It is tempting to speculate that ethanol may act on similar cellular targets in different species. Comparing the ethanol's effects on heterosexual courtship and copulation, chronic ethanol has opposite effects on sexual arousal and performance of male flies, indicating that their underlying processes may be distinct. It is possible that adaptive changes induced by chronic ethanol exposure are necessary to enhance sexual arousal and may overlap with those underlying behavioral sensitization on disinhibited courtship.

In summary, recurring ethanol administration has diverse effects on sexual behavior of *Drosophila* males including disinhibited intermale courtship, enhanced sexual arousal toward females and decreased sexual performance. We have identified three cellular components Fru^M^, White and dopamine that are crucial for the ethanol-induced courtship disinhibition. These findings support the notion that alcohol-associated sexual behavior is physiological and provides a baseline to further clarify the underlying cellular mechanisms.

## Materials and Methods

### Drosophila strains and culture

All fly stocks were reared on standard cornmeal medium at 25° C with 50% relative humidity under the 12h light/12h dark illumination condition. Isogenic *w^1118^*, *fru^3^*, and UAS-GFP lines were obtained from the Bloomington Stock Center and *f06905* from the Harvard Exelixis stock collection. *w^1^*, PHSBJb_3_, *fru^1^*, *TH-GAL4*, *DDC-GAL4*, and *UAS-Shi^ts^* lines were kindly provided by Drs. Ordway (PSU), Odenwald (NIH), Hall (Brandeis U.), Birman (Institute of Marseille), Hirsh (U. Virginia), and Kitamoto (U. Iowa), respectively. f06905 and PHSBJb_3 _males, which were backcrossed with *w^1118^* for five generations, were crossed with *w^1118^* females to produce *w^1118^;;mw^+^/+* and *w^1118^;;hs-mw^+^/+* males, respectively, and UAS-Shi^ts^ females were crossed with TH-GAL4, DDC-GAL4, and *f06905* to generate TH-GAL4/UAS-Shi^ts^, DDC-GAL4/UAS-Shi^ts^, and *mw^+^*/UAS-Shi^ts^, respectively, for behavioral tests. In addition, TH-GAL4 is crossed with UAS-GFP to produce TH-GAL4/UAS-GFP for visualizing dopamine neurons after chronic ethanol exposure. Males were collected within one or two days after eclosion and 33 males representing a group were housed together in a food vial before and between ethanol treatments.

### Behavioral assays

All ethanol exposures were performed in Flypub at room temperature (23°C) except for the Shi^ts^ experiments, which were conducted at 32°C (see below). Flypub was made of a plastic chamber (57 mm D×103 mm H) with a clear ceiling for videotaping behavior and an open bottom for administering ethanol. A group of 4 to 5 d-old (unless otherwise indicated) males was gently transferred into the chamber and the bottom was covered with Kimwipes. After the flies were acclimated to the chamber for 10 min, the whole unit was gently placed on top of a Petri dish (35 mm D) containing a cotton pad freshly applied with 1 ml of water, 70% ethanol (70% Flypub) or 95% ethanol (95% Flypub). Four to six Flypubs were recorded together using a digital video camera (PV-GS55, Panasonic Co., NJ) into Windows media movie files to monitor courtship activities. For the experiments performed under darkness, Sony HAD CCD camera with IR LEDs (Avalonics, NY, USA) was used for recording. Ethanol exposure was terminated when all flies were sedated and flies were kept in food vials till the next exposure on the following day.

To measure the sedating effect of ethanol, the number of flies lying on their back or immobile for over 10 sec at the bottom of the chamber was counted every 3 min. To obtain mean sedation time (MST), the number of sedated flies at a given time multiplied by the time of sedation was added up, which was then divided by the total number of flies. Courtship activities were monitored during 30 sec periods and the maximum number of flies engaged in courtship at a given time was scored. The average of 10 consecutive periods was used to represent the percentage of males engaged in courtship for each group. Sporadic courtship pairs that were occasionally formed and lasted for only a couple of seconds were not included. The experiments involving more than one genotype were carried out blindly to the experimenters who administered ethanol and scored courtship.

In the pheromone test, the chronic-ethanol-treated *CS* (daily exposure to ethanol vapor in 95% Flypub for 5 days) or age-matched ethanol-naïve *CS* males were decapitated on ice 1 h before ethanol treatment. To keep the decapitated males away from the bottom in Flypub, nylon mesh was inserted at 20 mm below the top of the chamber. The equal numbers (20 to 22) of decapitated ethanol-naïve males and intact chronic-ethanol-treated males, or decapitated chronic-ethanol-treated males and intact ethanol-naïve males, were transferred to the top compartment in Flypub. After 10 min of acclimation, the males were exposed to ethanol vapor in 95% Flypub and the total number of intact males courting decapitated males was counted every min. The average number of 5 consecutive min was used to yield the percentage of intact males courting decapitated males.

For testing dopamine's role in the ethanol-induced courtship, TH-GAL4/UAS-Shi^ts^, DDC-GAL4/UAS-Shi^ts^, and *mw^+^*/UAS-Shi^ts^ males were kept at 23°C before and between ethanol treatments. In the 32°C incubator with 50% relative humidity, the males were acclimated for 10 min and then exposed to ethanol vapor in Flypub containing a 50% ethanol pad. Under this condition, the males showed the sedation time comparable to that in 95% Flypub. The courtship activities were recorded and scored from the recorded movie files as described above.

For testing courtship and copulation of a male and female mixed population, the wings of either males or females were cut off on ice to distinguish the sex 1 h before ethanol exposure. Flypub was divided into two compartments using a filter paper to separately house males and females during acclimation. *CS* males (20 males per group) were exposed to daily ethanol in 95% Flypub for 5 days to represent chronic-ethanol-treated males. Age-matched ethanol-naïve or chronic-ethanol-treated males were transferred to one compartment in Flypub and the equal number of virgin *CS* females to the other compartment. After 10 min of acclimation, the filter paper was taken out to allow males and females to mix together immediately after the flies were exposed to ethanol vapor in 95% Flypub. Independent sets of age-matched ethanol-naïve males mixed with females were tested in Flypub without ethanol to score basal courtship and copulation activities.

Statistical analysis was performed using Minitab 14 (Minitab Inc., State College, PA, USA). Two-tailed Student's *t*-test was used to compare the means of two groups. When there were more than two groups, analysis of variance (ANOVA) and general linear model with *post hoc* Tukey-Kramer tests were used. All data are reported as mean±standard error of the mean.

### Ethanol assay

*CS* males (20 per group) were exposed to ethanol vapor in 95% Flypub for 1, 2, or 6 days. Sixteen or 30 min after the onset of ethanol exposure, the flies were frozen in liquid nitrogen and homogenized in 300 mL of 50 mM Tris pH 7.5 at 4°C. After 20 min of centrifugation, supernatants were used for the ethanol assay. The alcohol assay kit containing alcohol dehydrogenase and NAD (N7160, Sigma-Aldrich, St. Louis, MO) was dissolved in 0.5 M glycine solution, pH 9.0, containing 0.1 M hydrazine and used for ethanol measurements according to the manufacturer's instructions. To calculate molarities, the water content of each male was approximated to 0.65 mL [56].

## Supporting Information

Movie S1 Ethanol-induced intermale courtship in CS males. CS males were subjected to daily ethanol treatment in 95% Flypub. The QuickTime movie clip shows approximately 20 to 30 sec recordings of pre-, 1st and 6th exposures.
(2.71 MB MOV)

Movie S2 Ethanol-induced intermale courtship in w and transgenic w males. w and hs-mw+/+ males were subjected to daily ethanol exposure in 95% Flypub. The QuickTime movie clip shows approximately 20 to 30 sec recordings of the 6th exposure.
(2.29 MB MOV)
